# The Effects of as-Needed Nalmefene on Patient-Reported Outcomes and Quality of Life in Relation to a Reduction in Alcohol Consumption in Alcohol-Dependent Patients

**DOI:** 10.1371/journal.pone.0129289

**Published:** 2015-06-08

**Authors:** Clément François, Nora Rahhali, Ylana Chalem, Per Sørensen, Amandine Luquiens, Henri-Jean Aubin

**Affiliations:** 1 H. Lundbeck A/S, Issy les Moulineaux Cedex, France; 2 H. Lundbeck A/S, 2500 Valby, Denmark; 3 Assistance Publique-Hôpitaux de Paris, Hôpital Paul Brousse, INSERM U669, Villejuif Cedex, France; Mayo Clinic, UNITED STATES

## Abstract

**Background:**

The objective of this article was to investigate the effect of as-needed nalmefene on health-related quality of life (HRQoL) in patients with alcohol dependence, and to relate changes in drinking behavior and status to HRQoL outcomes.

**Methods:**

This *post hoc* analysis was conducted on a pooled subgroup of patients with at least a high drinking risk level (men: >60 g/day; women: >40 g/day) who participated in one of two randomized controlled 6-month studies, ESENSE 1 and ESENSE 2. Patients received nalmefene 18 mg or placebo on an as-needed basis, in addition to a motivational and adherence-enhancing intervention (BRENDA). At baseline and after 12 and 24 weeks questionnaires for the Medical Outcomes Study (MOS) 36-item Short-Form Health Survey (SF-36), European Quality of life-5 Dimensions (EQ-5D) and the Drinker Inventory of Consequences (DrInC-2R) were completed.

**Results:**

The pooled population consisted of 667 patients (nalmefene: 335; placebo: 332), with no notable between-group differences in baseline patient demographics/characteristics. At week 24, nalmefene had a superior effect compared to placebo in improving SF-36 mental component summary scores (mean difference [95% CI], p-value: 3.09 [1.29, 4.89]; p=0.0008), SF-36 physical component summary scores (1.23 [0.15, 2.31]; p=0.026), EQ-5D utility index scores (0.03 [0.00, 0.06]; p=0.045), EQ-5D health state scores (3.46 [0.75, 6.17]; p=0.012), and DrInC-2R scores (-3.22 [-6.12, 0.33]; p=0.029). The improvements in SF-36 mental component summary scores at week 24, and the DrInC-2R total score change from baseline to week 24, were significantly correlated to reductions in heavy drinking days and total alcohol consumption at week 24.

**Conclusions:**

As-needed nalmefene significantly improved almost all patient-reported HRQoL measures included in SF-36 and EQ-5D compared with placebo. These HRQoL gains were significantly correlated to reduced drinking behavior, as determined by reductions in heavy drinking days and total alcohol consumption.

## Introduction

Alcohol dependence has been estimated to affect 8 million persons in the USA [1] and 14.6 million in Europe [2] and, as such, is a major public health concern. It places enormous pressure on families, healthcare systems and society which increases with increasing alcohol consumption [3–5]. Alcohol is one of the most important avoidable risk factors for global burden of disease and disability. For example, in 2004, 3.8% of all global deaths (6.3% for men and 1.1% for women) and 4.6% of disease burden and injury (7.6% for men and 1.4% for women) were attributed to alcohol [6]. These rates are similar to those associated with the use of tobacco [6].

The traditional goal for the management of alcohol dependence has been abstinence, and this has been the primary endpoint in many clinical trials. However, less than 10% of patients with alcohol use disorders are undergoing treatment and the unwillingness to engage in an abstinence programme is a major roadblock to therapeutic success [7]. The availability of treatments aimed at reducing alcohol intake is likely to increase the proportion of patients entering treatment programmes and is increasingly being accepted as a viable therapeutic strategy [7–12].

Patients with alcohol dependence have lower levels of quality of life (QoL) compared with general population norms and compared with other chronic health conditions such as diabetes and heart failure [13–15]. Additionally, frequent or episodic heavy drinking patterns in non-dependent individuals are associated with reduced QoL compared with the general population. Importantly, regarding the clinical management of alcohol-dependent individuals, it has been shown that marked reductions in drinking, without complete abstinence, are associated with significant improvement in QoL [13–16].

A number of studies have evaluated health-related QoL (HRQoL) as the primary outcome measure in treatment trials of alcohol-dependent patients, generally with abstinence as the treatment goal [15–21]. In particular, the Medical Outcomes Study (MOS) 36-item Short-form Health Survey (SF-36) and the European Quality of life-5 Dimensions (EQ-5D) instruments have been used widely in clinical trials in many disease settings, including alcohol use disorders [22]. Acamprosate plus social support produced a general improvement in all SF-36 dimensions and summary scores after 3 months, with a further slight improvement after 6 months in a study involving alcohol-dependent patients [18]. Another study evaluated changes in HRQoL in alcohol-dependent patients entering a 3-week inpatient treatment program and found that scores for the eight individual SF-36 dimensions, as well as the mental and physical summary scores, had improved significantly by the end of treatment compared with baseline [15]. Frischknecht and colleagues reported that improved drinking behavior was associated with a better HRQoL after 7-years’ post-treatment follow-up in alcohol-dependent patients who underwent an initial 6-week inpatient and 1-month outpatient treatment program aimed at abstinence [16]. In a recent study in adults with alcohol dependence, a combination of medical treatment (naltrexone, acamprosate or disulfiram) with cognitive behavioral therapy was shown to reduce symptoms of depression and improve patients’ HRQoL as measured with EQ-5D. In particular, at 12, 52 and 119 weeks, treatment was associated with significant improvements from baseline for the dimensions of sleep, action, pain, and mood EQ-5D [21].

Nalmefene is an opioid system modulator with antagonistic activity at the μ and δ receptors, and partial agonist activity at the κ-receptor [23]. It appears to restore the balance of a dysregulated motivational system by reducing the reinforcing effect of alcohol, and thereby reducing the urge to drink alcohol. Treatment with nalmefene on an as-needed basis reduces total alcohol consumption and the number of heavy drinking days compared with placebo, as was shown in the two controlled 6-month efficacy studies, ESENSE 1 and ESENSE 2 [24,25]. Pooled data from ESENSE 1 and 2 involving alcohol-dependent patients with at least a high drinking risk level (as defined by the World Health Organization (WHO) [3]), demonstrated that nalmefene was associated with a superior effect compared with placebo in reducing the number of heavy drinking days and total alcohol consumption at month 6 [26]. Nalmefene was recently granted market approval in the European Union for the reduction of alcohol consumption in adult patients with alcohol-dependence who have at least a high drinking risk level according to the WHO (>60 g/day for men and >40 g/day for women [3]) and who continue to have a high drinking risk level 2 weeks after initial assessment [27].

This article presents a *post hoc* analysis of the high drinking risk alcohol-dependent sub-group from ESENSE 1 and 2. This sub-group was selected since a large improvement in drinking behavior was observed in both trials for the up to 2 weeks during the period between screening and randomization [24,25]. This phenomenon has been reported previously and means that a substantial fraction of the patients would be treated without a prospect of further improvement [26]. The primary aim of this analysis was to investigate the effect of as-needed nalmefene on HRQoL and a measure of the adverse consequences of alcohol abuse as assessed by patient-reported outcomes, and to relate changes in drinking behavior and status to HRQoL outcomes.

## Methods

Full details of the methods employed in ESENSE 1 and 2 have been published previously [24–26] and below is an overview to provide context for the reader.

### Study Design and Population

This analysis is based on two 6-month randomized, double-blind, placebo-controlled, efficacy studies, ESENSE 1 and ESENSE 2, which assessed nalmefene on an as-needed basis in a subgroup of patients with at least a high drinking level risk. The two studies were identical in design [24,25]. All patients took part in a motivational intervention programme (BRENDA) to support behavioral change and to enhance adherence to treatment [28].

Men and women aged ≥18 years with a primary diagnosis of alcohol dependence according to the Diagnostic and Statistical Manual of Mental Disorders [29], assessed with the Mini-International Neuropsychiatric Interview [30] were evaluated for eligibility at an initial screening visit. Both studies were conducted in accordance with the Declaration of Helsinki and the ICH Harmonized Tripartite Guideline for Good Clinical Practice, and were approved by the ethics committees at each study site. All patients provided written informed consent.

### Study Procedures and Assessments

One to two weeks after screening, patients were randomized 1:1 to 24 weeks double-blind as-needed treatment with nalmefene 18 mg or placebo. Patients were instructed to take one tablet each day they perceived a risk of drinking alcohol (as-needed dosing), preferably 1–2 h prior to anticipated time of drinking, but otherwise as soon as drinking had started. No specific treatment goal was defined, i.e. both abstinence and a reduction in alcohol consumption were acceptable. Assessments of efficacy and safety have been published elsewhere [24–26]. The focus of this analysis was to relate changes in drinking behavior and status with a measure of alcohol-associated problems (Drinker Inventory of Consequences [DrInC-2R]) and to measures of HRQoL as assessed by patient-reported outcomes using two relevant instruments: the SF-36 and the EQ-5D questionnaires which were administered at baseline, week 12 and week 24.

#### SF-36

The SF-36 is a patient-reported outcome instrument that was developed as a generic measure of perceived health status [31]. The standard SF-36 is a self-rated questionnaire comprising 36 questions which generate 8-domain health profiles using 3- to 5-point Likert scales. These are: physical functioning, bodily pain, role limitations due to physical problems, general health perception, mental health, energy and vitality, role limitations due to emotional problems, and social functioning. The first four domains are aggregated to create the Physical Component Summary (PCS) and the second four domains combined to create the Mental Component Summary (MCS).

Scores for each domain are calculated by using scoring algorithms detailed in the user’s manual by summing the item responses in each domain and then into a 0–100 range for each of the eight domains [32]. These are then standardized using means and standard deviations for the general population. Higher scores correspond to better health status or well-being. Systematic comparisons of the SF-36 with other commonly-used HRQoL instruments indicate that the SF-36 includes eight of the most frequently measured health domains, indicating content validity and relevance as a generic HRQoL measure [13,33]. The SF-36 is the most extensively used instrument for determining generic patient-reported outcome measures in general and specific populations, including individuals with alcohol use disorders [13]. It has proven useful for comparing the burden of different diseases, differentiating the health benefits of different treatments and for screening individual patients. Studies have demonstrated the reliability and validity of the SF-36 in alcohol-dependent populations [18].

#### EQ-5D

The EQ-5D is a generic measure of HRQoL in which health status is defined in terms of 5 dimensions: mobility, self-care, usual activities, pain/discomfort and anxiety/depression [34,35]. Each dimension has three qualifying levels of response roughly corresponding to 'no problems', 'some difficulties/problems', and 'extreme difficulties'. EQ-5D defines a total of 243 theoretically possible unique health states. The 5 items allow the calculation of a utility index ranging between -0.594 (the worst) and 1 (the best) [35]. For the rating of own health state status on the vertical visual analogue scale, a higher scores means a better health state [36].

#### DrInC

The Drinker Inventory of Consequences (DrInC) is a self-administered questionnaire comprising 50 items [37]. It was designed to measure adverse consequences of alcohol abuse in five areas: physical, intrapersonal, social, interpersonal and impulse control. The scale provides a previous 3-month measure of adverse consequences, and scores range from 0 to 135. The higher the total DrInC score the greater the adverse consequences for the patient.

### Statistical Analyses

For the current subgroup analyses the target efficacy population comprised all randomized patients who had at least one valid post-baseline assessment of both co-primary efficacy variables (heavy drinking days and total alcohol consumption) and at least a high drinking risk level (men: alcohol consumption >60 g/day; women: alcohol consumption >40 g/day), as defined by the WHO [3] at both screening and randomization. The baseline for drinking variables was defined as the month preceding the screening visit. For all other variables, the baseline was defined as the assessment at the screening visit.

Analysis of the pooled subgroup of patients with at least a high drinking risk level assessed changes from baseline in patient-reported outcomes using a mixed model repeated measures analysis (MMRM). The MMRM model used observed data and included the baseline value as a covariate and country, sex, time in weeks (Weeks 12 and 24), and treatment as fixed effects. The baseline value-by-time interaction and treatment-by-time interaction were also included in the model, and an unstructured covariance matrix was used.

Pearson correlation coefficients were estimated for changes in alcohol consumption (number of heavy drinking days and total alcohol consumption, from van den Brink and colleagues [26]) vs. changes in patient-reported outcomes in both the nalmefene and placebo groups and they were adjusted for baseline values of patient-reported outcomes.

All statistical tests were two-sided and p values < 0.05 were considered to be statistically significant. The statistical software used was SAS, Version 9.2.

## Results

1322 patients were randomized to treatment and of these 667 patients (50.5%) had at least a high drinking risk level at both screening and randomization (target population): 335 patients in the nalmefene group and 332 patients in the placebo group. Details of the target population included in this analysis have been published previously and there were no notable differences between the nalmefene and the placebo groups (Table 1) [26]. The mean age at baseline was 48 years, two-thirds of the patients were males, the cohort was almost exclusively Caucasian (99%) and the mean age at the onset of problem drinking (defined as drinking that starts to adversely affect an individual’s personal or professional life, or when the individual loses control over their drinking) was 35 years. Mean BMI at baseline was 26 kg/m^2^, and one-third of patients had previously been treated for alcohol dependence and 16% for alcohol withdrawal symptoms (Table 1). Mean (± standard deviation) baseline HRQoL scores on the SF-36 and EQ-5 dimensions surveys, and DrInC questionnaire scores were virtually identical in the nalmefene and placebo groups: SF-36 MCS score (40.9 ± 12.6 vs. 40.9 ± 12.6), SF-36 PCS score (50.2 ± 8.0 vs. 50.8 ± 8.0), EQ-5D utility index score (0.78 ± 0.21 vs. 0.80 ± 0.21), EQ-5D health state score (67.9 ± 17.2 vs. 68.9 ± 17.7), and DrInC total score (41.5 ± 22.0 vs. 41.3 ± 22.1), respectively.

**Table 1:** Baseline characteristics/demographics, including patient-reported outcomes, in patients with a high drinking risk level (target efficacy population) [adapted from [27]].

	High drinking risk level at screening and randomization (target population)		
	Placebo (n = 332)	Nalmefene (n = 335)	TOTAL (n = 667)
Race, Caucasian	329 (99.1%)	333 (99.4%)	662(99.3%)
Sex, Male	216 (65.1%)	223 (66.6%)	439(65.8%)
Age (years)	48.7 (10.5)	48.4 (10.5)	48.5 ± 10.5
Body Mass Index (kg/m2)	26.1 (4.4)	26.0 (4.8)	26.0 ± 4.6
Age at onset of problem drinking (years)	35.1 (11.6)	35.6 (12.3)	35.3 ± 11.9
Total monthly heavy drinking days (days)	22.4 (6.0)	22.9 (5.9)	22.6 ± 5.9
Total alcohol consumption (g/day)	103.3 (44.5)	107.7 (45.5)	105.5 ± 45.0
Clinical Global Impression—Severity of Illness	4.3 (1.4)	4.3 (1.4)	4.3 ± 1.4
Drinker inventory of consequences (DrInC) total score	42.2 (22.2)	41.1 (22.3)	41.6 ± 22.2
Alcohol dependence scale total score	13.3 (5.7)	14.0 (6.0)	13.7 ± 5.8
Current smoker	192 (57.8%)	184 (54.9%)	376(56%)
Living alone	99 (29.8%)	88 (26.3%)	187(28%)
Previously treated for alcohol dependence	112 (33.7%)	105 (31.3%)	217(32.5%)
Previously treated for alcohol withdrawal symptoms	59 (17.8%)	49 (14.6%)	108(16.2%)
Family history of alcohol problems	209 (63.0%)	211 (63.0%)	420(63.0%)

Data are mean (SD) or number of patients (%).

SD, standard deviation.

### Patient-Reported Outcomes

**Fig 1:**
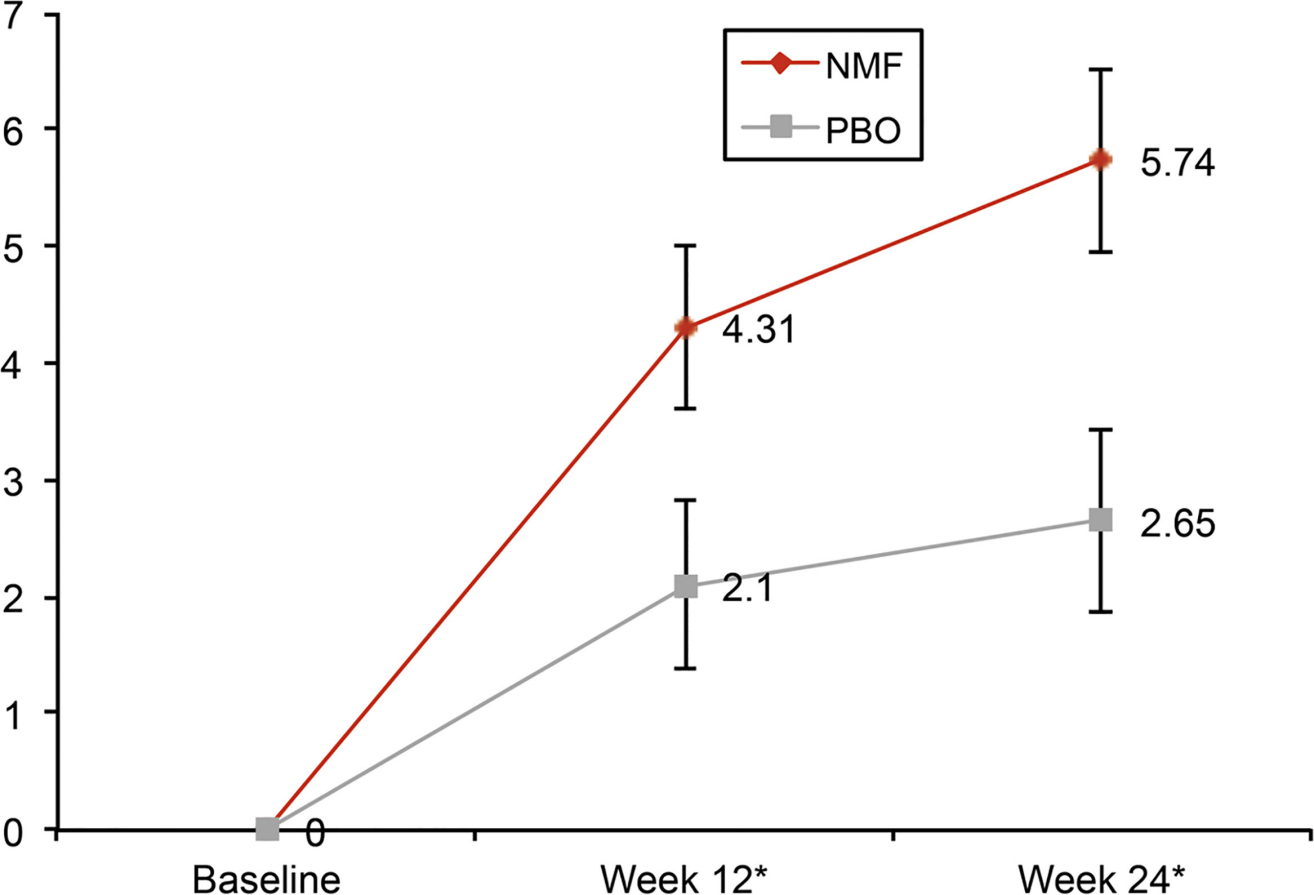
Adjusted mean change from baseline in SF-36 MCS Scores (mean ± SE). NMF, nalmefene; PBO, placebo; SF-36 MCS, Short-Form health survey-36 mental component summary. * p<0.05.

**Fig 2:**
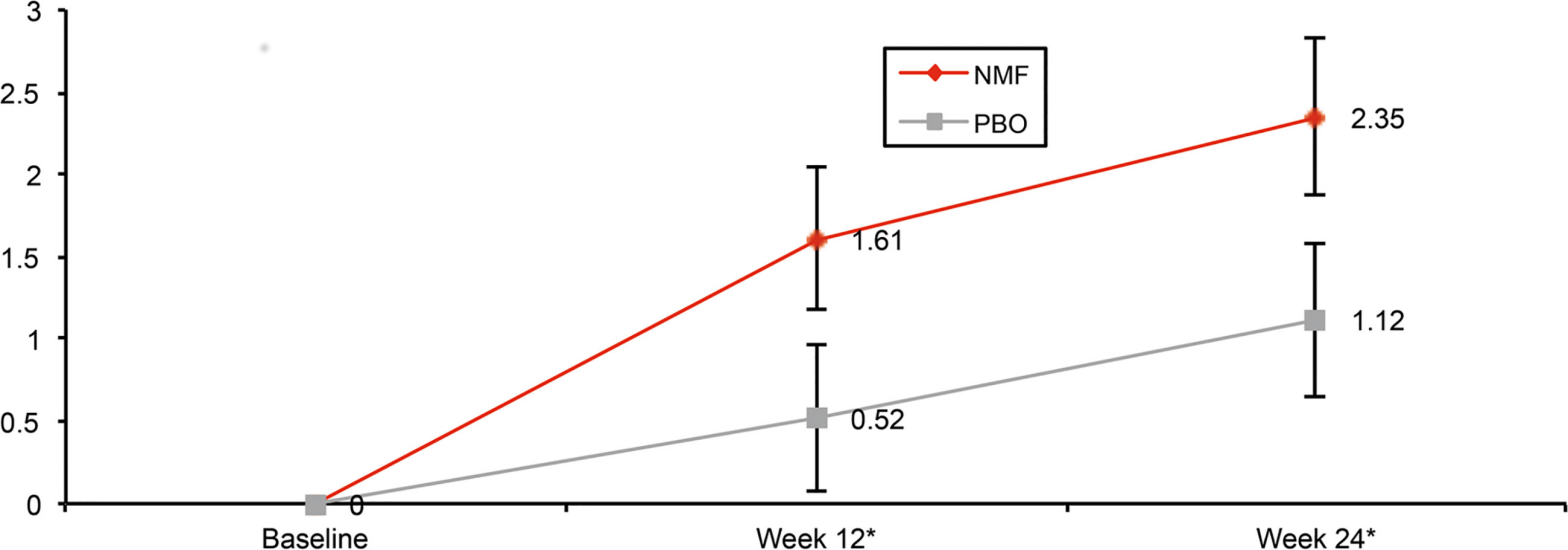
Adjusted mean change from baseline in SF-36 PCS Scores (mean ± SE). NMF, nalmefene; PBO, placebo; SF-36 PCS, Short-Form health survey-36 physical component summary. * p<0.05.

Using a MMRM analysis in the target efficacy population, nalmefene was shown to significantly improve SF-36 MCS, SF-36 PCS, EQ-5D utility index score and EQ-5D health state score after 12 and 24 weeks of treatment (Table 2). The changes from baseline in SF-36 MCS and SF-36 PCS were significantly greater for nalmefene versus placebo by week 12 and were further improved by week 24 as shown in Figs 1 and 2. The improvement in SF-36 MCS at week 24 was correlated to reductions in heavy drinking days (r = -0.1941; p<0.0001) and total alcohol consumption (r = -0.2061; p<0.0001) (Table 3).

**Table 2:** Adjusted Mean changes in SF-36, EQ-5D and DrInC scores from baseline to week 12 and week 24 in patients with a high drinking risk level (MMRM, target efficacy population).

		Change from baseline to Week 12		Difference to Placebo baseline to Week 12			Change from baseline to Week 24		Difference to Placebo baseline to Week 24		
		N	Mean ± SE	Mean ± SE	95% CI	p-value	N	Mean ± SE	Mean ± SE	95% CI	p-value
SF-36 MCS score	PLA	268	2.10 ± 0.73				218	2.65 ± 0.78			
	NMF	275	4.31 ± 0.70	2.21 ± 0.78	[0.68; 3.74]	0.0047	184	5.74 ± 0.79	3.09 ± 0.92	[1.29; 4.89]	0.0008
SF-36 PCS score	PLA	268	0.52 ± 0.45				218	1.12 ± 0.47			
	NMF	275	1.61 ± 0.43	1.09 ± 0.48	[0.15; 2.03]	0.0232	184	2.35 ± 0.48	1.23 ± 0.55	[0.15; 2.31]	0.0259
EQ-5D Utility Index score	PLA	278	0.01 ± 0.01				222	0.03 ± 0.01			
	NMF	279	0.04 ± 0.01	0.03 ± 0.01	[0.01; 0.06]	0.0185	188	0.06 ± 0.01	0.03 ± 0.02	[0.00; 0.06]	0.0445
EQ-5D Health State score	PLA	274	2.43 ± 1.10				221	3.13 ± 1.19			
	NMF	276	5.18 ± 1.07	2.75 ± 1.17	[0.45; 5.05]	0.0191	189	6.60 ± 1.20	3.46 ± 1.38	[0.75; 6.17]	0.0124
DrInC Total score^a^	PLA	280	-11.95 ± 1.23				226	-14.64 ± 1.30			
	NMF	282	-13.25 ± 1.20	-1.30 ± 1.31	[-3.86; 1.26]	0.3200	189	-17.86 ± 1.31	-3.22 ± 1.47	[-6.12; -0.33]	0.0292

^a^ A lower score indicates fewer alcohol-related problems

**DrInC**, drinker inventory of consequences; **EQ-5D**, EuroQoL-5 dimensions; **MCS**, mental component summary; **MMRM** = Mixed Model Repeated Measures; **NMF** = nalmefene; **PCS**, physical component summary; **PLA** = placebo; **SF-36**, Short-Form health survey-36.

**Table 3:** Correlation coefficients for the relationship between changes from baseline to Week 24 in the number of monthly heavy drinking days (HDD) and monthly total alcohol consumption (TAC) *versus* changes in patient-reported outcome measures in the total population (nalmefene and placebo pooled data).

	HDD		TAC	
	Pearson Correlation Coefficient^a^	p-value	Pearson Correlation Coefficient^a^	p-value
SF-36 MCS score	-0.1941	<0.0001	-0.2061	<0.0001
SF-36 PCS score	-0.0569	0.2578	-0.0707	0.1596
EQ-5D Utility Index score	-0.0772	0.1217	-0.0761	0.1271
EQ-5D Health State score	-0.2023	<0.0001	-0.1811	0.0003
DrInC Total score	0.2046	<0.0001	0.2446	<0.0001

^a^ negative values indicate a positive correlation for QoL while positive values indicates a positive correlation for DrInc

**DrInC**, drinker inventory of consequences; **EQ-5D**, EuroQoL-5 dimensions; **MCS**, mental component summary; **PCS**, physical component summary; **SF-36**, Short-Form health survey-36; **VAS**, visual analogue scale.

In the nalmefene group, SF-36 scores for all dimensions with the exception of ‘role physical’ were significantly improved from baseline to week 24 compared with placebo (Table 4). ‘Role physical’ exhibited a trend towards improvement (mean ± standard deviation difference vs. placebo 1.36±0.71, p = 0.0561).

**Table 4:** Adjusted mean change from baseline to week 24 in SF-36 dimensions in patients with a high drinking risk level (MMRM, target efficacy population).

Short-Form health survey-36 (SF -36) Dimensions		Baseline		Change from Baseline to Week12		Difference to Placebo			Change from Baseline to Week 24		Difference to Placebo		
		N	Mean ± SD	N	Mean ± SE	Mean ± SE	95% CI	p-value	N	Mean ± SE	Mean ± SE	95% CI	p-value
Bodily Pain	Placebo	319	49.16 ± 10.66	279	0.73 ± 0.67				226	1.47 ± 0.70			
	Nalmefene	316	49.85 ± 10.63	283	2.46 ± 0.66	1.73 ± 0.74	[0.28; 3.17]	0.0195	189	3.90 ± 0.72	2.43 ± 0.82	[0.82; 4.05]	0.0032
General Health	Placebo	318	43.84 ± 9.56	277	0.85 ± 0.56				224	1.46 ± 0.61			
	Nalmefene	318	44.71 ± 9.53	283	2.47 ± 0.54	1.62 ± 0.60	[0.45; 2.79]	0.0066	189	3.82 ± 0.62	2.36 ± 0.71	[0.96; 3.76]	0.0010
Mental Health	Placebo	319	41.97 ± 11.35	280	2.01 ± 0.66				226	1.82 ± 0.70			
	Nalmefene	316	41.66 ± 11.34	283	2.01 ± 0.66	1.61 ± 0.71	[0.21; 3.01]	0.0247	188	4.99 ± 0.72	3.17 ± 0.83	[1.54; 4.79]	0.0001
Physical Functioning	Placebo	317	50.95 ± 6.84	277	0.50 ± 0.43				223	1.03 ± 0.43			
	Nalmefene	318	51.01 ± 7.01	281	1.59 ± 0.42	1.09 ± 0.47	[0.16; 2.02]	0.0216	187	2.24 ± 0.44	1.21 ± 0.49	[0.24; 2.18]	0.0142
Role Emotional	Placebo	316	42.36 ± 11.40	274	0.67 ± 0.71				221	2.33 ± 0.75			
	Nalmefene	317	42.46 ± 11.46	280	3.25 ± 0.69	2.58 ± 0.77	[1.06; 4.10]	0.0009	188	4.66 ± 0.77	2.33 ± 0.89	[0.58; 4.08]	0.0093
Role Physical	Placebo	316	45.51 ± 9.2	273	0.94 ± 0.56				222	2.00 ± 0.60			
	Nalmefene	317	45.89 ± 9.34	281	2.64 ± 0.54	1.71 ± 0.60	[0.52; 2.89]	0.0048	188	3.36 ± 0.61	1.36 ± 0.71	[-0.04; 2.75]	0.0561
Social Functioning	Placebo	319	43.33 ± 11.95	280	3.93 ± 0.61				226	3.20 ± 0.67			
	Nalmefene	317	44.32 ± 10.62	284	2.09 ± 0.63	1.84 ± 0.69	[0.49; 3.19]	0.0078	189	5.36 ± 0.68	2.17 ± 0.79	[0.62; 3.71]	0.0061
Vitality	Placebo	319	47.04 ± 10.16	280	1.96 ± 0.60				226	1.97 ± 0.65			
	Nalmefene	319	47.48 ± 9.56	283	3.55 ± 0.58	1.59 ± 0.64	[0.33; 2.86]	0.0135	188	4.30 ± 0.66	2.34 ± 0.77	[0.83; 3.84]	0.0024

MMRM: mixed model repeated measures.

EQ-5D utility index scores were improved to a significantly greater extent by nalmefene compared with placebo after 12 (p = 0.0185) and 24 (p = 0.0455) weeks (Table 2). Likewise, EQ-5D health state scores were also improved to a significantly greater extent by nalmefene compared with placebo after 12 (p = 0.0191) and 24 (p = 0.0124) weeks (Table 2). There was a significant correlation between improvement in EQ-5D health state scores and a reduction in the number of heavy drinking days (r = -0.2023; p < 0.0001) and also with a reduction in total alcohol consumption (r = -0.1811; p = 0.0003) (Table 3). No such relationships were observed for EQ-5D utility index scores and changes in heavy drinking days and total alcohol consumption.

DrInC-2R total scores were improved to a greater extent by nalmefene than placebo, which is indicative of fewer alcohol-related problems, and the mean [95% CI] difference at 24 weeks was statistically significant (-3.22 [-6.12, 0.33]; p = 0.0292). The improvement in DrInC-2R total scores were significantly correlated to both a reduction in heavy drinking days (r = 0.2046; p < 0.0001) and total alcohol consumption (r = 0.2446; p < 0.0001) (Table 3).

Despite a reduction in the number of patients assessed at baseline, week 12 and week 24, there were no differences in baseline characteristics between patients who withdrew and patients who completed each study. Baseline characteristics and response to treatment for patients who withdrew and patients who completed each study were similar. Thus, there was no apparent missing-data mechanism. This applies to both the total population and to our *post-hoc* analysis population.

## Discussion

The current *post hoc* analysis was designed to establish whether improvements in drinking behavior were associated with benefits in patient-reported outcomes for HRQoL and fewer alcohol-related problems. The assessment of HRQoL among alcohol-dependent subjects presents a number of challenges in the absence of a specific instrument developed specifically for alcohol dependence [13,22]. In this analysis we employed two instruments to monitor patient-reported outcomes related to HRQoL: SF-36 and EQ-5D. Compared with placebo, nalmefene produced significant improvements in HRQoL as assessed by patient-reported outcomes such as: SF-36 MCS, SF-36 PCS, 7 of 8 dimensions in the SF-36, EQ-5D utility index score and EQ-5D health state score. The only measure which did not achieve statistical significance was the ‘role physical’ dimension in SF-36 (mean difference versus placebo 1.36; p = 0.0561). At baseline the mean SF-36 MCS was 40.9 in both the placebo and nalmefene groups and this is markedly lower than the reference value for the U.S. general population. Interestingly, SF-36 PCS was over 50 at baseline in the two groups indicating that alcohol-dependent patients perceived their problems to be more psychological than physical. A similar finding was reported by Daeppen and colleagues in an early study using SF-36 to evaluate HRQoL in alcohol- dependent patients [38].

This analysis of patients with a high drinking risk level has confirmed significant patient-reported benefits in HRQoL with as-needed nalmefene in a controlled clinical comparison with placebo. Minimally Important mean group Differences (MIDs) in SF-36 scores (MCS, PCS, and the 8 domains) between the nalmefene and placebo groups were determined to assess whether the results were clinically meaningful. In our study the following MIDs were recorded: 3 for MCS, and mental health; and 2 for role-emotional, bodily pain, general health, social functioning and vitality. The greater improvements in HRQoL, with MIDs of 2–4, are clinically relevant from the patients’ perspective [32]. Further, the improvement in EQ-5D utility index score for nalmefene versus placebo was similar to that observed in an observational longitudinal study in patients with alcohol dependence who shifted to lower alcohol consumption and fewer alcohol-related problems [20]. Unfortunately, the lack of published data regarding MIDs for the DrInC-2R scale precluded our ability to definitively associate clinical relevance to improved DrInC-2R scores. Nevertheless, the improvements in HRQoL found in the current study parallel significant reductions in heavy drinking days and total alcohol consumption reported in the same cohort [26].

This analysis also established a significant correlation between a reduction of alcohol intake (heavy drinking days and total alcohol consumption) and an improvement of HRQoL measures such as SF-36 MCS and EQ-5D health state scores, as well as total DrInC scores. A similar finding was previously reported in a naltrexone trial [39]. Even though the causal nature of the relationship between alcohol drinking reduction and HRQoL improvement cannot be inferred from the results of our study, this finding is an important contribution to the rationale for the “reduction concept” as a goal, in addition to the abstinence goal in the management of alcohol dependence. The benefits in HRQoL are in addition to the reported reductions in all-cause mortality resulting from decreased alcohol consumption in heavy drinkers (defined as at least 60 g of alcohol per day in men or 40 g alcohol per day in women) [40,41].

This study has a number of limitations. Firstly, it was a *post hoc* analysis of patients with a high drinking risk level, rather than the total randomized population. However, as noted by van den Brink and colleagues, this reflects the behavior of patients pre-randomization, and the nalmefene and placebo groups were comparable in terms of numbers and baseline demographics/characteristics [26]. These patients are by definition at greater risk of alcohol-related harm and therefore represent the patients in most need of treatment. Another potential limitation relates to the original ESENSE 1 and 2 selection criteria which excluded patients with significant DSM-IV axis I co-morbidity and serious withdrawal symptoms, although this is in line with EMA guidelines for the development of pharmacological products for the treatment of alcohol dependence [10]. Other limitations of our study relate to the patient-reported outcomes used to gauge treatment effects on HRQoL. For example, the SF-36 questionnaire was administered every 3 months during the study; HRQoL may deteriorate during the first few weeks of treatment, and this would have been missed in the current study. In addition, SF-36 does not monitor problems with sleeping which can have a detrimental effect on HRQoL in alcohol-dependent individuals [18]. Furthermore, while SF-36 has been shown to be useful in alcohol-dependent subjects [38,42] it is nonetheless a generic health status measure, and is not based on specific symptoms associated with alcohol dependency. The information it collects may not be completely relevant or specific for alcohol-dependent subjects and it may not be the ideal HRQoL measure in this patient group [22]. However, as yet, no specific instrument has been developed to assess changes in HRQoL in alcohol-dependent patients [22]. With this in mind, a new HRQoL instrument which is specific to alcohol use disorder, namely the Alcohol Quality of Life Scale (AQoLS), is being developed and validated [22]. This instrument is currently available in English, French and Japanese language versions. Nevertheless, despite any potential shortcomings with using the generic SF-36 in patients with alcohol dependence, the HRQoL improvements observed in nalmefene-treated patients were significantly better than those achieved with placebo.

In conclusion, this analysis from two controlled clinical trials (ESENSE 1 and 2) demonstrated that nalmefene as-needed improved almost all patient-reported measures of HRQoL included in SF-36 and EQ-5D. These HRQoL gains paralleled significantly reduced drinking behavior as determined by reductions in heavy drinking days and total alcohol consumption, as well as fewer alcohol-related problems (as assessed by total DrInC-2R scores) [24–26]. While longer term experience is clearly needed, the move towards pharmacologically supported reductions in alcohol consumption, reduced health risk and improved HRQoL are highly desirable. Reduction rather than abstinence may be a more palatable treatment goal for patients with alcohol dependence, and an advance in this complex and difficult to manage therapeutic setting.
